# Development of the Bisphenol A exposure scale in adults

**DOI:** 10.3389/fpubh.2024.1504189

**Published:** 2024-11-27

**Authors:** Betül Kaplan, Tülay Ortabağ, Ekrem Aslan

**Affiliations:** ^1^Department of Medical Services and Techniques, Hasan Kalyoncu University, Gaziantep, Türkiye; ^2^Department of Nursing, İstanbul Topkapı University, İstanbul, Türkiye; ^3^Abdülkadir Yüksel State Hospital, Gaziantep, Türkiye

**Keywords:** Bisphenol-A, scale, Bisphenol-A exposure, good health and well-being, responsible consumption and production

## Abstract

**Objective:**

This study was conducted to develop a scale for assessing the attitudes of adults regarding the determination of Bisphenol A exposure.

**Methods:**

The study sample comprised of 370 individuals who volunteered to participate. According to the Explanatory Factor Analysis (EFA) results of the investigation, a scale structure consisting of a total of 3 sub-dimensions was obtained. In the Confirmatory Factor Analysis, the scale item factor loading values were acceptable.

**Results:**

The fit indices for the scale were CMIN/df = 1,618, RMSEA = 0.058, NFI = 0.914, CFI = 0.965, and IFI = 0.790, indicating a satisfactory level of agreement. The scale was determined to have a Cronbach value of 0.79 and a high degree of reliability. The item-total score correlation coefficients of the scale ranged from 0.327 to 0.534 and exhibited a high degree of discrimination, as determined.

**Conclusion:**

Based on the analyses conducted, it was determined that the Adult Bisphenol A Exposure Scale is a valid and reliable instrument for determining the attitudes of adults toward contact with and use of Bisphenol A-containing products.

## 1 Introduction

Environmental pollutants are a significant problem in the globe. Bisphenol A (BPA), one of these substances, is a chemical found in plastics, storage containers, packaging, and a variety of other everyday items. It has been suggested that this substance may have a negative effect on the hormonal system and lead to early puberty, obesity, behavioral issues, and fetal sex difficulties. Although BPA has an estrogenic effect as one of the endocrine disrupting chemicals and is reported to have less effect than other endocrine disruptors, its negative effects on human health are significant due to its widespread use in industry and everyday life. Long-term exposure to endocrine-disrupting chemicals (EDC) has been shown to be associated with metabolic dysfunction, reproductive system disorders, endocrine-related malignancies, and neurodevelopmental disorders (1). EDCs are environmental compounds that have the potential to disrupt the endocrine system of humans and wildlife (1). Numerous studies have demonstrated that long-term exposure to these chemicals may be associated with metabolic dysfunction, reproductive system disorders, endocrine-related malignancies, and neurodevelopmental disorders in humans (1–6). Bisphenol-A is the most prevalent EDC due to its ubiquitous utilization. As one of the pollutants that attract the most attention and have the greatest potential to imperil human and environmental health, it is regarded as a significant public health issue of which society should be aware.

BPA was first synthesized by Aleksandr P. Dianin in 1891, and its potential commercial use was investigated during the 1930s search for synthetic estrogen. In the 1940s and 1950s, the plastics industry identified BPA’s uses (7). Bisphenol A (BPA) is produced by the condensation of phenol and acetone (8) in the presence of an acid or alkaline compound. BPA is soluble in all organic solvents and partially in water. At ambient temperature, it exists as a white solid particle or crystal (7). It can bind to the estrogen receptor (ER) like estradiol and exert both estrogenic and anti-androgenic properties (9, 10). These properties are related primarily to the 4-hydroxyl group on the N-phenyl ring and the hydrophobic moiety at the 2-position of the propane moiety (9). Since the majority of BPA-containing products come into contact with food, this is crucial for human health and the environment (11).

BPA is used in the production of certain plastics and compounds. It is present in polycarbonate (PC) plastics, which are frequently utilized in food and beverage containers such as water bottles and other consumer goods. Epoxy resin, which is used to coat the interior of metal products such as food cans, also contains BPA (12). Humans can be exposed to BPA through multiple routes, including transdermal, oral, and inhalation. Major sources of BPA exposure include packaged foods, thermal paper, infant items, pollution, medical equipment, and dental materials, among others (13). Humans are most frequently exposed to BPA through tinned foods and beverages. BPA leaches from canned foods and beverages. Environmental conditions, such as high temperature, sunlight, and acidic tinned foods such as tomatoes, exacerbate this absorption to the point where it seeps into the food through the can linings. Everyday activities, such as using plastic implements to microwave food and storing plastic beverage bottles in heated vehicles, also increase BPA leaching from plastics into food (14, 15). Scientists and the general public have begun to express concern due to the expanding use of Bisphenol A (BPA) in a variety of applications and the mounting evidence of its endocrine-disrupting effects (16).

People are primarily exposed to BPA through food (17). However, given that natural foods that are expected to be BPA-free, as well as edible animals and animal products, are grown in a polluted and hazardous environment, it can be predicted that the problem will not be limited to plastic, tinned, or ready-to-eat foods. Tons of BPA are used annually in numerous industrial sectors, and it disperses into the environment and atmosphere. In groundwater near waste sites contaminated with BPA-containing substances, a higher concentration of BPA and accumulation of plastic detritus have been discovered (18).

The majority of people today are employed and consume ready-made goods. Even though people have the ability to protect themselves from BPA, they continue to unknowingly use products containing BPA. However, no scale evaluating the use of plastic packaged products by adults and Bisphenol A exposure information has been identified in the literature. In order to eliminate this deficiency in the scientific field, it is believed that the construction of a scale that evaluates the use of plastic packaged products and Bisphenol A exposure information will contribute to the literature.

## 2 Methods

This is a methodological study conducted to ascertain the exposure status of adults to Bisphenol A. The design of the scale consisted of four stages: scale development, factor structure, reliability and validity assessment.

### 2.1 Phase 1. Scale’s development

Following an exhaustive literature evaluation, a pool of questions containing suggestions regarding the use of plastic packaged products was compiled. After constructing the pool of scale items, expert opinions on content validity were obtained. The received expert opinions were evaluated using the Lawshe method (19). After the survey was completed, its language was evaluated, and any necessary adjustments were made. In accordance with expert opinions, the number of items in the scale was reduced to 37. The invariance of the scale over time was evaluated by administering it a second time to 50 academicians and administrative personnel who were not included in the study group 4 weeks after the initial administration, and calculating the correlation between the scores obtained in the two administrations. In addition to the “Adult Bisphenol A Exposure Scale,” the study’s questionnaire included inquiries about the sociodemographic characteristics (age, gender, occupation) of the participants.

### 2.2 Phase 2. Factorial structure

Using Exploratory Factor Analysis (EFA), the purpose of this phase is to evaluate the factor structure. Additionally, the internal consistency of the factors was analyzed using Reliability Analysis.

#### 2.2.1 Participants

In this study, there was no sample selection to determine the sample size; instead, the sample size was determined by the requirement that the number of samples be five to 10 times the average number of items on the scale in scale development studies (20). It was 35.38 ± 10.03 years. 34.6% of the participants were between the ages of 29 and 34, 51.6% were male, and 55.1% were administrative staff. In addition, researchers were given an informed consent form prior to the study. Volunteer researchers participated in the study, while participants with insufficient data were excluded.

#### 2.2.2 Data analysis

In our research, the elements were initially constructed by the researcher based on a review of the relevant literature. Then, the scale was constructed, factors were extracted utilizing Exploratory Factor Analysis, and the consistency of the factors was examined utilizing Reliability Analysis. The research data was analyzed using the IBM SPSS 25 bundle program and AMOS 24 software. Participants’ data were summarized using mean, standard deviation, percentage, and frequency distributions. The initial stage involved the calculation of the correlation matrix. The Bartlett and Kaiser Meyer Olkin (KMO) tests were then computed to determine if the data were suitable for factor analysis and if each item contained the necessary assumptions for this analysis and subsequent tests (21, 22). Then, to construct a conceptual model, “Principal Axis Factoring” was selected as the method, and “promax” was used to conduct factor analysis. After this step, the internal consistency (Cronbach’s alpha) of each factor’s items was determined.

### 2.3 Phase 3: Reliability, validation of the Bisphenol A exposure scale in adults

**Figure 1:**
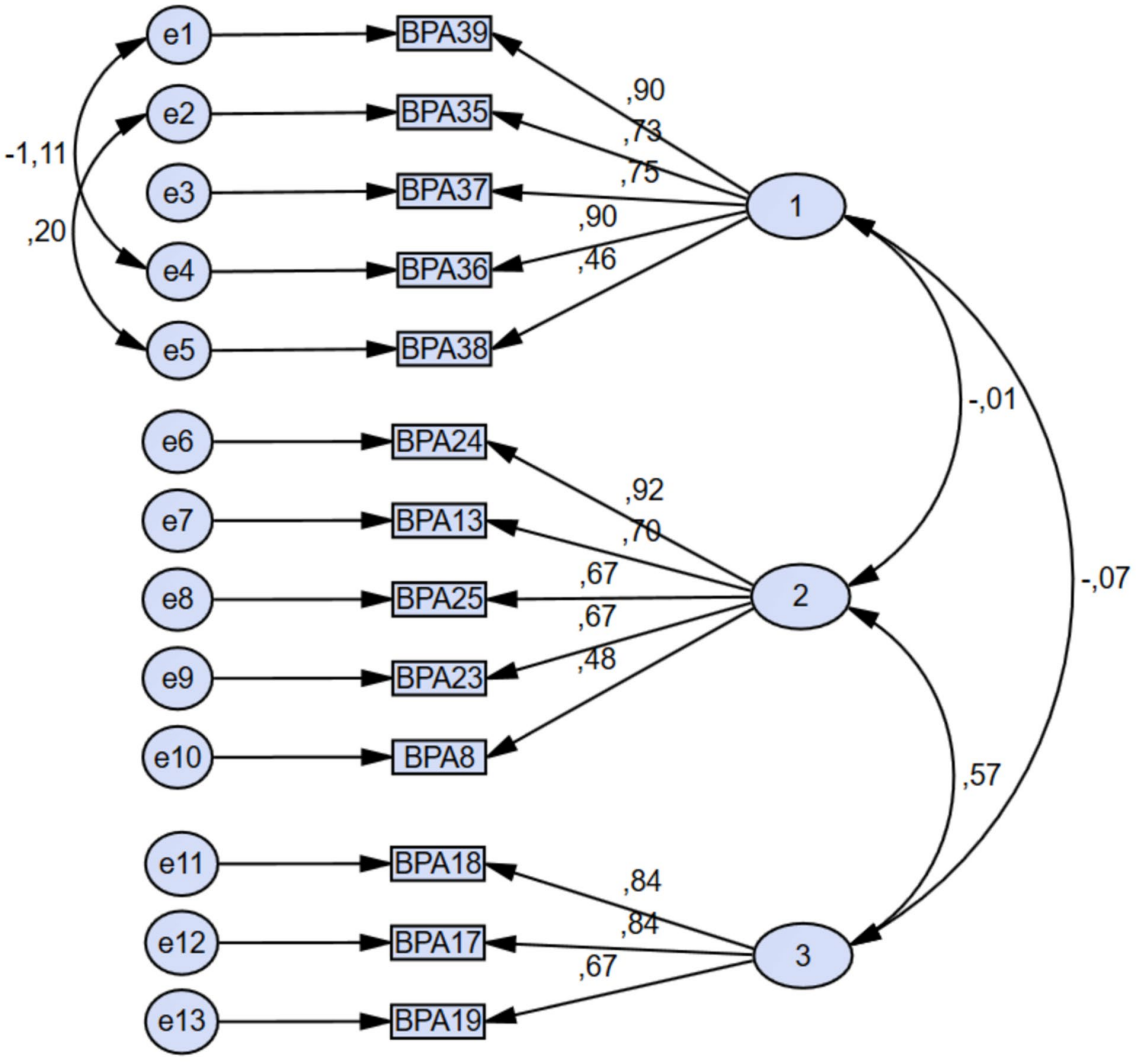
Confirmatory factor analysis fit indices and model view.

Confirmatory Factor Analysis (CFA) is a statistical method used to evaluate the fit between theoretical constructs and measurement models and to verify their validity. Model fit indices derived from the CFA method are used to assess the accuracy of the EFA results on a comparable sample data set collected by the researcher and to evaluate the scale’s validity (Figure 1). As the estimation point for CFA, the Maximum Likelihood method was used. This technique is often used to enhance the normal distribution assumption, parameter estimation, and fit indices. Diverse methodologies were used to evaluate the scale’s reliability: The item-total score correlation, internal consistency (Cronbach), test–retest, and upper-lower 27% discrimination procedures were utilized. Intraclass correlation and Pearson correlation analysis were applied to the test–retest procedure. Construct validity was determined using Exploratory and Confirmatory Factor Analysis (EFA and CFA, respectively). Principal axis factorization and promax rotation were favored throughout the EFA phase. In determining the number of factors, only those variables with eigenvalues of one or greater were considered. By using the method of determining the variance according to the explanation (contribution) rate, a variance rate between 40 and 60% was considered sufficient. The chi-square value *p* > 0.05 was used as a criterion for assessing the CFA model’s quality of fit. In addition, NNFI (Non-normed Fit Index), NFI (Normed-Fit Index), CFI (Comparative Fit Index), and RMSEA (Root Mean Square Error of Approximation) fit indices were determined. As criteria for acceptable levels of fit indices, NNFI and CFI > 0.95, NFI > 0.90, and RMSEA<0.05 were used. At a significance level of *p* < 0.05, the obtained study results were evaluated.

## 3 Results

Bisphenol A Exposure Scale in Adults; Bartlett’s sphericity test was statistically significant (x^2^ = 1197.172, *p* < 0.001), and Kaiser-Meyer-Olkin (KMO = 0.81) was greater than 0.60. Since the individual KMO values of the 3rd, 11, 28, and 29th items on the scale were less than 0.60, they were excluded from the study to preserve the integrity of the analysis, in accordance with the literature. These two experiments demonstrated that factor analysis is applicable to this scale. As a result of the EFA, “Communality” values were discovered first. Communality is a value that measures the relationship between the items and the factors, and a value below 0.40 is not desirable. Items numbered 2, 4, 6, 7, 9, 10, 12, 15, 16, 20, 22, 30, 31, 32, 33, and 34, as well as items 21 and 27, are also eliminated from the scale because they coincide. Removed. It was observed that there were a total of three factors. Factor 1 is comprised of five items with factor loadings ranging from 0.531 to 0.848, and its contribution to the total variance explained is 29.7%. Factor 2 consists of five items with factor loadings ranging from 0.574 to 0.974; its contribution to the total variance explained is 21.9%. Factor 3 comprises of three items with factor loadings ranging from 0.549 to 1.011; its contribution to the total variance explained is 7.2%. The calculated total explained variance rate was 58.9% (Table 1).

**Table 1:** Bisphenol A exposure scale explanatory factor loadings for adults.

Item no	Items	Factors and factor loadings		
		1	2	3
39	Do you use cosmetic products such as lipstick, blush, foundation?	0.848		
35	Do you use sun protection cream?	0.800		
37	Do you use hair dye, hair styler, conditioner for cosmetic purposes?	0.793		
36	Would you use a face mask for cosmetic purposes?	0.774		
38	Do you use perfume, deodorant, roll-on for cosmetic purposes?	0.531		
24	Do you consume soft drinks such as soda, fizzy drinks, and fruit juice in plastic packages?		0.974	
13	Do you consume canned drinks at home or outside?		0.771	
25	Do you consume drinks with plastic straws?		0.667	
23	Do you consume products such as milk, cream and kefir in plastic packages?		0.584	
8	Do you drink hot liquid foods in plastic cups?		0.574	
18	Can you use vinegar in plastic containers?			1.011
17	Do you use oils in plastic containers?			0.670
19	Would you consume pickles in plastic containers?			0.549
Eigenvalues		4.243	3.234	1.244
Total variance explained (%)		29.681	21.968	7.246
Cumulative variance explained (%)		29.681	51.649	58.895
Kaiser Meyer Olkin (KMO) and Bartlett Test Results				
Bartlett test		KMO	0.815	
		*ꭓ^2^*	1197.172	
		sd	78	
		*p*	<0.001	

Extraction method: Principal axis factoring, Rotation method: Promax.

### 3.1 Introductory information of the participants

The 370 participants in the study ranged in age from 23 to 73, with an average age of 35.38 ± 10.03 years. 34.6% of the participants were between the ages of 29 and 34, 51.6% were male, and 55.1% were administrative staff (Table 2).

**Table 2:** Distribution of descriptive characteristics of participants (*n* = 370).

Variables	Mean ± Sd	Min. – Maks.
Age	35.38 ± 10.03	23–73
Variables and Subgroups	Frequency (n)	%
Age categories		
23 to 28 years	90	24.4
29 to 34 years	128	34.6
35 to 40 years	86	23.2
41 and older	66	17.8
Gender		
Female	179	48.4
Male	191	51.6
Position		
Academician	166	44.9
Administrative personnel	204	55.1
Total	370	100.0

SD, Standard deviation; Min., Minimum; Maks., Maximum.

### 3.2 Validity findings

#### 3.2.1 The validity of scope and content

In order to develop the “Bisphenol A Exposure Scale in Adults,” 9 experts with a high level of knowledge and experience in the field (three professors, two associate professors, one lecturer, and one public health nurse) evaluated a pool of 40 questions. The “Expert Evaluation Form” was sent to the experts’ email addresses so they could submit their evaluations. Using the Lawshe method, the data from the expert evaluation form were analyzed. While the Content Validity Rate (CVR) was determined for each item, the Content Validity Index (CVI) was determined for the complete scale form. Each item’s CVR rate should ideally be positive (+) and near to 1. If the values derived from expert opinions are 0 or negative (−), the question in query pool should be eliminated.

In a new scale to be developed using the Lawshe method, it is anticipated that the CVR for each item and the CVI for the total number of items will exceed the value calculated based on the number of experts in the Lawshe content validity criterion values Table (23) (19). The value corresponding to nine experts in the Lawshe content validity criterion Table is 0.778 in this instance. In accordance with the advice of experts, items with scores below 0.778 were eliminated from the query pool. In this regard, expert opinions determined that the 5, 14, and 26th items did not meet the content validity criterion. CVR and CVI were recalculated after removing these objects. The entire CVI value was determined to be 0.93 by the Lawshe method.

#### 3.2.2 Construct validity

Statistical analysis techniques including factor analysis, internal consistency analysis, and hypothesis testing are used to examine construct validity (24). EFA and CFA were conducted within the scope of this study to ascertain construct validity. For the construct validity analysis of the Bisphenol A Exposure Scale in Adults, it was first determined whether or not each item was appropriate for Exploratory Factor Analysis (EFA). KMO and Bartlett tests were used in this regard. To evaluate the sample size, a KMO value of 0.815% was calculated. This result suggests the sample size is adequate for EFA. The calculated result of the Bartlett test was ꭓ^2^ = 1197.172, p0.001. Therefore, this result indicates whether the correlation coefficients between the items are suitable for EFA.

Prior to examining the results of this analysis, the KMO values of each sample item were evaluated. Since the individual KMO values of the 3rd, 11, 28, and 29th items on the scale were less than 0.60, they were excluded from the study to preserve the integrity of the analysis, in accordance with the literature. The eigenvalue coefficient is utilized when determining or separating factors. In general, factorization occurs when this value is 1 or greater. The number of factors in the scale, eigenvalue coefficients, explained variances, and factor loadings of each item in terms of the factor it is under are enumerated in Table 1.

Figure 1 depicts the use of model fit indices derived from the CFA method to test the accuracy of the EFA results on an identical sample data set collected by the researcher and to assess the validity of the scale. Maximum Likelihood method was used as the estimation point in CFA.

In our research, CMIN/DF (ꭓ^2^ = 97.069 / df = 60, *p* = 0.002) = 1.618 (Chi-square / degrees of freedom) value, RMSEA = 0.058 (Root Mean Square Error of Approximation), GFI = 0.930 (Goodness of Fit Index), AGFI = 0.894 (Adjusted Goodness of Fit Index), NFI = 0.914 (Normed Fit Index), NNFI (TLI) = 0.954 (Non- Normed Fit Index), CFI = 0.965 (Comparative Fit Index), IFI = 0.965 (Incremental Fit Index) fit index values show that the model has a good fit. To enhance the AGFI and NFI values, a modification (adjustment) was applied between items 35 and 38 and between items 36 and 39.

The sub-dimension factor loadings ranged from 0.46 to 0.90 for Factor 1, 0.48 to 0.92 for Factor 2, and 0.67 to 0.84 for Factor 3 (Figure 1).

### 3.3 Reliability results

In order to test the reliability of the scale within the scope of this study, the frequently used item sub-dimension and item total score analyses, Cronbach reliability coefficient, lower-upper 27% comparison (item discrimination), and test–retest methods for invariance over time were selected.

Bisphenol A Exposure Risk Scale sub-dimension item correlation analysis results (Corrected Item-Total Correlation) are given in Table 3.

**Table 3:** Item-subdimension and total score correlations for the Bisphenol A exposure risk scale for adults (*n* = 370).

All items		Adjusted	
		Sub-dimension item total score correlation	Item total score correlation
		r	r
1.	Have you heard of a chemical called BPA or Bisphenol?	Not included	Not included
Factor 1			
39.	Do you use cosmetic products such as lipstick, blush, foundation?	0.753	0.343
35.	Do you use sun protection cream?	0.724	0.355
37.	Do you use hair dye, hair styler, conditioner for cosmetic purposes?	0.729	0.431
36.	Would you use a face mask for cosmetic purposes?	0.727	0.478
38.	Do you use perfume, deodorant, roll-on for cosmetic purposes?	0.495	0.400
Factor 2			
24.	Do you consume soft drinks such as soda, fizzy drinks, and fruit juice in plastic packages?	0.511	0.534
13.	Do you consume canned drinks at home or outside?	0.678	0.490
25.	Do you consume drinks with plastic straws?	0.587	0.475
23.	Do you consume products such as milk, cream and kefir in plastic packages?	0.812	0.479
8.	Do you drink hot liquid foods in plastic cups?	0.602	0.345
Factor 3			
18.	Can you use vinegar in plastic containers?	0.681	0.430
17.	Do you use oils in plastic containers?	0.752	0.415
19.	Would you consume pickles in plastic containers?	0.577	0.327
40.	Have you had a dental filling?	Not included	Not included

The Adult Bisphenol A Exposure Scale was created as a 5-point Likert scale. When the distributions of the scale total and sub-dimensions are examined, namely “always = 0,” “often = 1,” “sometimes = 2,” “rarely = 3” and never = 4″, the lowest value that can be derived from the scale is 0, the lowest value that can be obtained from the scale is 0. The highest value found in this study was 52, and the average score was 24.74 ± 9.37. The Cronbach reliability coefficient for the full scale was calculated to be 0.79.

Comparing the difference between the item total score averages for the Lower and Upper 27% groups yields statistically significant information regarding the internal consistency of the scale as well as the item validity. A total of 370 individuals who participated in the study had their average scores ranked from highest to lowest. According to the calculation, there should be 100 individuals in each of the upper and lower 27% sections. According to the results of the independent samples t test conducted between the two groups for the scale total, the upper 27% group’s average score was higher than the lower 27% group’s average score, and the difference was statistically significant (*t* = 36.589, SD: 198, *p* < 0.001). Similar outcomes were observed for variables 1, 2, and 3. Accordingly, it can be stated that the distinctive characteristics of the items on the total scale and all subfactors, as well as the measurement capability of the measuring instrument in terms of internal consistency, are all high (Table 4).

**Table 4:** Test–retest analysis results based on intra-class correlation coefficient concerning the reliability of the Bisphenol A exposure scale in adults.

		%95		F test			
		Confidence interval		Statistics value			
		Lower limit	Upper limit		df1	df2	*p*
Single measurements	0.909	0.843	0.947	21.727	49	49	<0.001
Average measurements	0.952	0.915	0.973	21.727	49	49	<0.001

df, degrees of freedom.

The analysis determined that the intra-class correlation coefficient is 0.952. This value indicates that the scale’s reliability is outstanding (Table 4).

The homogeneity of the participants’ responses to the scale elements was evaluated using Hotelling’s T2 test. As determined by the analysis, Hotelling’s T2 = 727.446; *F*(12, 358) = 58.813, *p* 0.001. Based on this result, it can be concluded that the scale does not contain any response bias.

Table 5 presents the final version of the Bisphenol A Exposure Scale in Adults, reflecting the comprehensive adjustments and validations made throughout the study.

**Table 5:** Final version of the Bisphenol A exposure scale in adults.

Previous number	Last number	Questions
No.	No.	
1.	1	Have you heard of a chemical called BPA or Bisphenol?
Factor 1: Personal BPA Exposure		
39.	2	Do you use cosmetic products such as lipstick, blush, foundation?
35.	3	Do you use sun protection cream?
37.	4	Do you use hair dye, hair styler, conditioner for cosmetic purposes?
36.	5	Would you use a face mask for cosmetic purposes?
38.	6	Do you use perfume, deodorant, roll-on for cosmetic purposes?
Factor 2: BPA Exposure in the Home Environment		
24.	7	Do you consume soft drinks such as soda, fizzy drinks, and fruit juice in plastic packages?
13.	8	Do you consume canned drinks at home or outside?
25.	9	Do you consume drinks with plastic straws?
23.	10	Do you consume products such as milk, cream and kefir in plastic packages?
8.	11	Do you drink hot liquid foods in plastic cups?
Factor 3: BPA Exposure to Shopping Attitudes		
18.	12	Can you use vinegar in plastic containers?
17.	13	Do you use oils in plastic containers?
19.	14	Would you consume pickles in plastic containers?
40.	15	Have you had a dental filling?

## 4 Discussion

In this research, a scale was created to assess adult Bisphenol A exposure. The adult Bisphenol A exposure scale consists of three factors and 15 items. The first factor is “Personal BPA exposure” with 5 items, the second is “BPA exposure related to the home environment” with 5 items, and the third is “BPA exposure related to shopping attitude” with 3 items. Scale items 1 and 15 will not be evaluated. The gauge was constructed using a 5-point Likert scale. Participants’ responses were scored as follows: “always = 0″, “often = 1″, “sometimes = 2″, “rarely = 3″, and “never = 4” The scale’s minimum value is 0 and its maximum value is 52, while the average score for this study was 24.74 ± 9.37. A high score indicates a minimal exposure to Bisphenol A. The Cronbach reliability coefficient for the full scale was calculated to be 0.79. Test–retest is one of the methods for analyzing the reliability of an instrument. This test provides information regarding the scale’s internal consistency (25, 26). As its name suggests, test–retest is founded on measurements taken at specific periods or time intervals. It is suggested that this duration be between 4 and 6 weeks. In test–retest scale investigations, two distinct test results are typically analyzed for reliability (26). Reporting any one of these results is sufficient. The Pearson Product Moment Correlation Coefficient and the Intraclass Correlation Coefficient (ICC) are examples.

Pearson correlation is a statistical technique used to determine the relationship between scores obtained when the same individuals take the same test multiple times. This correlation is used to assess the precision and dependability of the measuring instrument. Because the Pearson correlation coefficient value indicates the extent of the relationship between two measurements.

ICC is a statistical method that is most commonly employed in research designs to evaluate the scope and consistency of a measurement instrument across repeated measurements (25, 27). ICC indicates the repeatability of measurements by the measuring instrument. ICC has a value between 0 and 1, and a value of 0.70 or higher indicates that the measurement instrument is reliable and yields stable results in repetitive measurements (28). ICC’s computation procedure evaluates 10 distinct hypotheses. It is essential to indicate which of these techniques was employed (27).

In reliability studies, ICC is more effective than Pearson correlation analysis because it incorporates both the correlation between measurements and the agreement between absolute results (27, 29). ICC, two-way mixed model with absolute fit was preferred for reliability in this analysis, contingent on the type of mean calculation. However, the study also included Pearson correlation analysis results in order to compare the two values. The analysis determined that the intra-class correlation coefficient is 0.952. This value indicates that the scale’s reliability is outstanding. When Test–Retest Analysis Based on Pearson Correlation Coefficient is applied regarding the reliability of the scale; It was determined that there was a positive, highly strong, statistically significant relationship between the first measurement and the second measurement (*r* = 0.912, *p* < 0.001). This correlation indicates the consistency and dependability of the scale over time.

### 4.1 Limitations and future research

These findings were derived from a convenience sample with limited generalizability. Academic and administrative personnel working at a foundation university were included in the sample. Consequently, the current scale will require additional research and the incorporation of various professional groups. Additionally, profession-specific research should be conducted. Also intriguing would be a study of factorial invariance by occupational category and associated socioeconomic status.

## 5 Limitations

The study has some limitations worth noting. It primarily involved academic and administrative personnel in Turkey, which may affect the generalizability of the findings to broader populations. Additionally, cultural and economic variations in different regions were not specifically addressed. The “BESA” scale is designed for adults and does not evaluate exposure in other age groups. Furthermore, reliance on self-reported data could influence the accuracy of the results. Acknowledging these factors can help guide future research and improve the scale’s applicability.

## 6 Conclusion

As a consequence, most people in modern society are involved in business and consume prepared foods. In addition, cosmetic products and plastic and resin-coated materials used in the home pose a high risk of Bisphenol A exposure. Despite the availability of BPA-protection options, people continue to unknowingly use BPA-containing products. No exposure measurement scale for Bisphenol A could be identified in the literature review. The validity and reliability of the scale indicate that it can be used to determine the exposure status of adults to Bisphenol A. In this regard, it is recommended that the Adult Bisphenol A Exposure Scale, which was developed as a result of this study, be utilized in scientific studies to ascertain in detail the issues and problems associated with adult Bisphenol A exposure.

Moreover, it is anticipated that the “Bisphenol A Exposure Scale in Adults (BESA) “will be applicable in various nations. However, this research was conducted with academic and administrative personnel from Turkey. Therefore, additional research is required to validate the scale in other nations.

## 7 Future implications

Based on the findings of this study, future research should focus on validating the Adult Bisphenol A Exposure Scale (BESA) in different cultural and demographic contexts to enhance its global applicability. Additionally, longitudinal studies could explore the long-term health impacts of Bisphenol A exposure, using the BESA to monitor exposure levels. This scale could also serve as a foundation for developing public health interventions aimed at reducing BPA exposure, particularly in high-risk populations such as those heavily reliant on packaged foods and plastic products.

## Data Availability

The raw data supporting the conclusions of this article will be made available by the authors, without undue reservation.

## References

[1] de Aguiar Greca S-C Kyrou I Pink R Randeva H Grammatopoulos D Silva E. Involvement of the endocrine-disrupting chemical Bisphenol a (Bpa) in human placentation. J Clin Med. (2020) 9:405. doi: 10.3390/jcm9020405, PMID: 32028606 PMC7074564

[2] Koch CA Diamanti-Kandarakis E. Introduction to endocrine disrupting chemicals–is it time to act? Rev Endocr Metab Disord. (2015) 16:269–70. doi: 10.1007/s11154-016-9338-3 26885651

[3] Yolton K Xu Y Strauss D Altaye M Calafat AM Khoury J. Prenatal exposure to Bisphenol a and phthalates and infant Neurobehavior. Neurotoxicol Teratol. (2011) 33:558–66. doi: 10.1016/j.ntt.2011.08.003, PMID: 21854843 PMC3183357

[4] Rahman MS Kwon W-S Lee J-S Yoon S-J Ryu B-Y Pang M-G. Bisphenol-a affects male fertility via fertility-related proteins in spermatozoa. Sci Rep. (2015) 5:9169. doi: 10.1038/srep09169, PMID: 25772901 PMC4360475

[5] Watkins DJ Sánchez BN Téllez-Rojo MM Lee JM Mercado-García A Blank-Goldenberg C. Phthalate and Bisphenol a exposure during in utero windows of susceptibility in relation to reproductive hormones and pubertal development in girls. Environ Res. (2017) 159:143–51. doi: 10.1016/j.envres.2017.07.051, PMID: 28800472 PMC5623649

[6] Sirasanagandla SR Al-Huseini I Sofin R Das S. Perinatal exposure to Bisphenol a and developmental programming of the cardiovascular changes in the offspring. Curr Med Chem. (2022) 29:4235–50. doi: 10.2174/0929867328666211206111835, PMID: 34872473

[7] Zielińska M Wojnowska-Baryła I Cydzik-Kwiatkowska A. Bisphenol a Removal from Water and Wastewater. Cham: Springer (2019).

[8] Shaw I. Endocrine-disrupting chemicals in food. Cambridge: Woodhead Publishing Limited (2009).

[9] Kitamura S Suzuki T Sanoh S Kohta R Jinno N Sugihara K. Comparative study of the endocrine-disrupting activity of Bisphenol a and 19 related compounds. Toxicol Sci. (2005) 84:249–59. doi: 10.1093/toxsci/kfi074, PMID: 15635150

[10] Sun H Xu L-C Chen J-F Song L Wang X-R. Effect of Bisphenol a, Tetrachlorobisphenol a and pentachlorophenol on the transcriptional activities of androgen receptor-mediated reporter gene. Food Chem Toxicol. (2006) 44:1916–21. doi: 10.1016/j.fct.2006.06.013, PMID: 16893599

[11] Michałowicz J. Bisphenol a–sources, toxicity and biotransformation. Environ Toxicol Pharmacol. (2014) 37:738–58. doi: 10.1016/j.etap.2014.02.003 24632011

[12] Monneret C. What is an endocrine disruptor? C R Biol. (2017) 340:403–5. doi: 10.1016/j.crvi.2017.07.004, PMID: 29126512

[13] Irshad K Rehman K Sharif H Tariq M Murtaza G Ibrahim M. Bisphenol a as an Edc in metabolic disorders. In: Endocrine disrupting chemicals-induced metabolic disorders and treatment strategies. Cham: Springer (2021). p. 251–63.

[14] Kabir ER Rahman MS Rahman I. A review on endocrine disruptors and their possible impacts on human health. Environ Toxicol Pharmacol. (2015) 40:241–58. doi: 10.1016/j.etap.2015.06.009 26164742

[15] Ain QU Roy D Ahsan A Farooq MA Aquib M Hussain Z. Endocrine-disrupting chemicals: occurrence and exposure to the human being. In: Endocrine disrupting chemicals-induced metabolic disorders and treatment strategies. Cham: Springer (2021) p. 113–23.

[16] Rochester JR Bolden AL. Bisphenol S and F: a systematic review and comparison of the hormonal activity of Bisphenol a substitutes. Environ Health Perspect. (2015) 123:643–50. doi: 10.1289/ehp.1408989, PMID: 25775505 PMC4492270

[17] Vandenberg LN Hauser R Marcus M Olea N Welshons WV. Human exposure to Bisphenol a (Bpa). Reprod Toxicol. (2007) 24:139–77. doi: 10.1016/j.reprotox.2007.07.010 17825522

[18] Kawagoshi Y Fujita Y Kishi I Fukunaga I. Estrogenic chemicals and estrogenic activity in leachate from municipal waste landfill determined by yeast two-hybrid assay. J Environ Monit. (2003) 5:269–74. doi: 10.1039/b210962j, PMID: 12729267

[19] Lawshe CH. A quantitative approach to content validity. Pers Psychol. (1975) 28:563–75. doi: 10.1111/j.1744-6570.1975.tb01393.x

[20] Şencan H. Reliability and validity in social and behavioral measurements. Ankara: Seçkin Publishing (2005).

[21] Arsham H Lovric M. Bartlett's Test. Int encyclopedia of statistical sci. (2011) 2:20–3. doi: 10.1007/978-3-642-04898-2

[22] George D Mallery P. Ibm Spss Statistics 27 Step by Step: A Simple Guide and Reference (17th Ed.). New York: Routledge (2021).

[23] Ayre C Scally AJ. Critical values for Lawshe’s content validity ratio: revisiting the original methods of calculation. Meas Eval Couns Dev. (2014) 47:79–86. doi: 10.1177/0748175613513808

[24] Büyüköztürk Ş Kılıç Çakmak E Akgün ÖE Karadeniz Ş Demirel F. Bilimsel Araştırma Yöntemleri [scientific research methods]. Ankara: PegemA (2008).

[25] Mehta S Bastero-Caballero RF Sun Y Zhu R Murphy DK Hardas B. Performance of Intraclass correlation coefficient (Icc) as a reliability index under various distributions in scale reliability studies. Stat Med. (2018) 37:2734–52. doi: 10.1002/sim.7679, PMID: 29707825 PMC6174967

[26] Weir JP. Quantifying test-retest reliability using the Intraclass correlation coefficient and the Sem. J Strength Cond Res. (2005) 19:231–40. doi: 10.1519/00124278-200502000-00038, PMID: 15705040

[27] Koo TK Li MY. A guideline of selecting and reporting Intraclass correlation coefficients for reliability research. J Chiropr Med. (2016) 15:155–63. doi: 10.1016/j.jcm.2016.02.012 27330520 PMC4913118

[28] Liljequist D Elfving B Skavberg RK. Intraclass correlation–a discussion and demonstration of basic features. PLoS One. (2019) 14:e0219854. doi: 10.1371/journal.pone.0219854, PMID: 31329615 PMC6645485

[29] Ulupinar S. Atletik Performans Ölçümlerinde test–Tekrar test Güvenirliği Analizleri. İstanbul Gelişim Üniversitesi Sosyal Bilimler Dergisi. (2022) 9:738–47. doi: 10.17336/igusbd.809612

